# Spontaneous Spiritual Awakenings: Phenomenology, Altered States, Individual Differences, and Well-Being

**DOI:** 10.3389/fpsyg.2021.720579

**Published:** 2021-08-19

**Authors:** Jessica Sophie Corneille, David Luke

**Affiliations:** ^1^Centre for Mental Health, School of Human Sciences, University of Greenwich, London, United Kingdom; ^2^Centre for Psychedelic Research, Department of Brain Sciences, Faculty of Medicine, Imperial College London, London, United Kingdom

**Keywords:** spirituality, mystical experience, altered states of consciousness, mental health, epilepsy, absorption, DMT, psilocybin

## Abstract

Spontaneous Spiritual Awakenings (SSAs) are subjective experiences characterised by a sudden sense of direct contact, union, or complete nondual merging (experience of oneness) with a perceived ultimate reality, the universe, “God,” or the divine. These profound transformative experiences have scarcely been researched, despite extensive anecdotal evidence suggesting their potential to catalyse drastic, long-term, and often positive shifts in perception, world-view, and well-being. The aims of this study were to investigate the phenomenological variances of these experiences, including the potential differences between SSAs and Spontaneous Kundalini Awakenings (SKAs), a subset of awakening experiences that the authors postulate may produce a higher likelihood of both physical and negative effects; to explore how these experiences compare to other altered states of consciousness (ASCs), including those mediated by certain psychedelic substances; and understand their impact on well-being. Personality trait absorption and temporal lobe lability (TLL) were assessed as predictors of Spontaneous Spiritual and Kundalini Awakenings (SSA/SKAs). A mixed within and between-participants self-report survey design was adopted. A total of 152 participants reporting their most powerful SSA/SKAs completed questionnaires measuring nondual, kundalini, and mystical experience, as well as depth of ASC, and trait absorption and TLL. Spontaneous Kundalini Awakenings were found to be significantly more physical, but not significantly more negative than SSAs, and overall, both sets of experiences were perceived to be overwhelmingly more positive than negative, even in cases where the experience was initially challenging. The phenomenological distribution of SSA/SKAs was similar to other measured ASCs although greater in magnitude, and appeared most similar in distribution and in magnitude to drug-induced ASCs, particularly classic psychedelics DMT and psilocybin. Temporal lobe lability and trait absorption were found to predict the SSA/SKA experience. The limitations and implications of these findings are discussed.

## Introduction

Spiritual awakening is a term given to describe a subjective experience in which an individual's ego transcends their ordinary, finite sense of self to encompass a wider, infinite sense of truth or reality. These deeply embodied, noetic experiences are often perceived as a direct connection, communion, or nondual merging with an unlimited and universal consciousness, the divine or “God” in perceived oneness (James, 1902/1985; Feuerstein, 1989; McClintock et al., 2016). Experiences of spiritual awakening, whether gradual or sudden, intentionally induced or spontaneous, typically evoke an ineffable sense of deep inner knowing, understanding, “remembering,” or “unveiling” of one's true nature, as well as experiences of peace and equanimity, bliss, ecstasy and aliveness, feelings of awe, sacredness, gratitude and reverence, and of abundant, unconditional love (James, 1902/1985; Stace, 1960; Pahnke and Richards, 1966; Hood, 1975; Lukoff et al., 1995; Griffiths et al., 2006, 2008, 2011; Taylor, 2012; Taylor and Egeto-Szabo, 2017). These profound experiences may also trigger a sense of transcendence of time and space, as well as an increase in physical and mental sensitivity to internal and external stimuli, including sensitivity to colour, light, touch, sounds, and smells (Hood, 1975; Taylor and Egeto-Szabo, 2017; Woollacott et al., 2020). In some cases, they may be accompanied by strong physical sensations, as appears to be more typical in what are usually referred to as *kundalini* awakenings, including but not limited to: sensations of heat or energy rising or “shooting up” in the body, typically in and around the spine; bursts of tingling, tickling, prickling in the body, particularly around the crown of the head, brow-point, and heart-space; electric sensations in the extremities of body; perceived light emanating from the body, particularly from the head and heart; orgasmic sensations; disruptions in the digestive system; and spontaneous involuntary movements, including trembling or shaking, *asanas* (yogic postures) and *mudras* (hand postures) (Ring and Rosing, 1990; Greyson, 1993; Greenwell, 2002; Taylor, 2013, 2015; Woollacott et al., 2020). Occasionally, these sensory sensitivities may extend to paranormal-like experiences, with people reporting increased synchronicities, visions of an archetypal or symbolic nature, telepathic experiences, feeling spiritual presences, hearing sounds or voices not produced externally, and seeing things that are not materially present (Greyson, 1993, 2000; Thalbourne and Fox, 1999; Sovatsky, 2009; Taylor, 2015; Grof, 2019).

Experiences referred to as kundalini awakenings were first highlighted in the tantric and yogic scriptures of fifth and sixth centuries AD, namely the *Yogavasishtha, Yoga Kundalini Upanishad*, and *Hatha Yoga Pradipika* (Taylor and Egeto-Szabo, 2017). These experiences were considered powerful catalysts to awaken latent potential through the unification of the polarities of the mind (perceived duality), into oneness (perceived nonduality). As seen through the lens of these traditions and the lineages that have followed, kundalini energy lies dormant, coiled at the base of the spine until it is moved, or awakened. As it uncoils, the energy makes its way up the central *naadi* (subtle energy channels) adjacent to the spine: namely the *ida* (left channel) and the *pingala* (right channel), through the *shushumna* (central channel), piercing each *chakra* (energy point) as it reaches *sahasrara* (the crown of the head). The settling of this energy at the crown of the head is said to provoke experiences of spiritual ecstasy, or enlightenment (Taylor, 2013, 2015; Taylor and Egeto-Szabo, 2017; Woollacott et al., 2020). Whilst both terms are interchangeable and clearly overlap, kundalini awakenings are typically associated with greater physical and energetic symptoms than general spiritual awakenings (Sanches and Daniels, 2008; De Castro, 2015; Lockley, 2019).

Traditional texts from the tantra and yoga lineages, such as the *Paratrisika Vivarana* and *Yoga Kundalini Upanishad*, discuss the attainment of spiritual and kundalini awakenings through *asana* (yogic movements), *mudra* (hand postures), *pranayama* (breathwork and breath retention), *bandhas* (body locks), and the manipulation of the *ojas* (libido energy) through the act of *brahmacarya* (celibacy or chastity) (Mallinson, 2011; Sovatsky, 2014; Parker, 2018). While recent studies confirm the power of spiritual contemplative practices such as yoga, meditation, and mindfulness as catalysts for gradual and sudden states of awakening (De Castro, 2015; Taylor and Egeto-Szabo, 2017), these may also occur during sex (Wade, 2001, 2004), near-death experiences (Rivas et al., 2016), contact with nature (Terhaar, 2009; Taylor, 2012), as a result of homeostatic imbalance (e.g., from fasting, sleep deprivation, or intense athletic activity) (Parry et al., 2007; Murphy and White, 2011), and through the use of mind-altering substances, particularly of classic psychedelics (Johnson et al., 2019) such as psilocybin, lysergic acid diethylamide (LSD), and *N,N-*dimethyltryptamine (DMT) (Griffiths et al., 2006, 2008, 2011; MacLean et al., 2011; Lyvers and Meester, 2012), although they appear to most frequently emerge following periods of prolonged psychological turmoil or trauma, including loss, bereavement, and addiction (Miller and C'de Baca, 2001; Taylor, 2012; Taylor and Egeto-Szabo, 2017). However, whilst specific conditions, factors and/or triggers may pre-empt these experiences, these may also occur void of any apparent trigger, though this appears to be less frequent (Miller and C'de Baca, 2001; Taylor, 2012; Taylor and Egeto-Szabo, 2017). Awakening experiences do not appear, therefore, to be mediated by the subject's spiritual or religious context (Perry, 1974; Lukoff, 1985; Taylor, 2012), as is further illustrated by the responses of 1,509 American participants to a Gallup survey from 2002, to which a staggering 41%—projecting to 80 million American adults—fully identified with the statement “I have had a profound religious experience or awakening that changed the direction of my life,” 25% of whom reported having no religious preference (Gallup, 2003). This response may also suggest that profound mystical experiences such as spiritual and kundalini awakenings might occur more frequently within the general population than generally considered (Lukoff, 1985).

Studies suggest that the peak duration of spiritual and kundalini awakenings is typically short, lasting from several minutes to several hours, with traces lingering for a longer period (Marshall, 2005). However, the very nature of one of its prominent features, the transcendence of time and space, may make it challenging for participants to recall the exact duration of the peak of their experiences. Whether long or short in peak duration, these intense psychological shifts in consciousness often lead to long-lasting and even permanent changes to the subject's sense of self and of the world around them (Neumann and Campbell, 1964; Taylor and Egeto-Szabo, 2017), often from an experience of fragmentation of purpose and meaning, to loving engagement with life (Dunnington, 2011). These experiences are therefore considered deeply healing. Some of the cognitive and behavioural shifts linked to these experiences include: increased empathy, compassion, gratitude, openness, trust, altruism, curiosity, awareness, creativity, authenticity, integrity, a sense of higher purpose and meaning in life, a sense of virtuous mission or selfless service towards humanity, a sense of being reborn and liberated from past attitudes and beliefs, a sense of devotion to love-based values, and a rejection of “religiousness” and materialistic lifestyles (Cook, 2004; McClintock et al., 2016; Taylor and Egeto-Szabo, 2017; McGee, 2020). These deep shifts may lead to radical changes in religious and philosophical views, relationships, and career paths (Taylor and Egeto-Szabo, 2017).

Spirituality may act as a buffer against stress and improves coping against the depressive effects of stressful events (Kendler et al., 1997), promoting positivity, equanimity, optimism, peace, and resilience (Grodzicki and Galanter, 2006; Brown et al., 2013). It is perhaps unsurprising, therefore, that spiritual and kundalini awakenings are associated with a wealth of sustained positive therapeutic outcomes, such as a decreased risk of committing suicide among suicidal individuals following their experience (Horton, 1973). Psilocybin-occasioned mystical experiences have been linked to sustained improvements in treatment-resistant depression (Carhart-Harris et al., 2016), and significant reductions in anxiety, hopelessness, and fear of death in patients with life-threatening cancer (Griffiths et al., 2016; Ross et al., 2016). Both psilocybin-occasioned mystical experiences and spiritual or kundalini awakenings attained without the use of drugs have been linked to persisting positive effects in the treatment of treatment-resistant alcohol and tobacco addiction (Green et al., 1998; Galanter et al., 2007; Strobbe et al., 2013; Garcia-Romeu et al., 2014), with several studies indicating a 3 to 4-fold increase in abstinence from addiction following spiritual awakening (Green et al., 1998; Kaskutas et al., 2003; Galanter et al., 2013). The very basis of the Alcoholics Anonymous programme lies in spiritual attainment, or awakening (Khouzam and Kissmeyer, 1997; Galanter, 2008; Strobbe et al., 2013)—even Jung proposed that spiritual awakening may enable healing from addiction (Allamani et al., 2013). Furthermore, these experiences may lead to an increased interest in spiritual based lifestyles associated with improved positive identity, positive coping, problem solving, and integrity (Taylor, 2012, Woollacott et al., 2020), which in turn have been linked to a decrease of psychopathological tendencies (McClintock et al., 2016).

In some cases, spiritual and kundalini awakenings trigger challenging short or long-term sensory, affective, cognitive, and physical effects (Neumann and Campbell, 1964; Grof and Grof, 2017; Woollacott et al., 2020) such as, but not limited to: panic, disorganised thoughts and behaviours, persistent involuntary movements of the body, uncomfortable sensations of heat and burning in the body, digestive problems, and challenging extrasensory-like experiences (Greyson, 1993; Taylor, 2015; Grof and Grof, 2017; Woollacott et al., 2020)—additionally, these experiences have been linked to a better performance in psi tasks involving precognition (Storm and Goretzki, 2020). Distressing awakening experiences, also known as *spiritual emergencies* or crises, may arise as a direct consequence of the initial experience, when an individual is left feeling overwhelmed, confused, or challenged by the drastic perceptual shifts that tend to emerge from these experiences, and by their potentially powerful energetic nature. Spiritual emergencies may also occur during the integration period following an awakening experience (Grof and Grof, 2017), as the subject finds themself stripped of all pre-existing beliefs and concepts of life without an appropriate framework through which to interpret their newly-gained insight, and often without an appropriate support system to which they can turn (Lukoff and Everest, 1985). Because of this, it is assumed that spiritual emergencies may more frequently occur in cases of sudden or *spontaneous* awakening (St Arnaud and Cormier, 2017), and outside of a religious or spiritual context (Taylor, 2013). However blissful initially, the experience of deep psychological change catalysed by spiritual or kundalini awakenings may provoke distress leading to spiritual emergency, which psychiatrists are likely to diagnose as acute psychotic experience indicative of psychopathology (Menezes and Moreira-Almeida, 2010; Grof and Grof, 2017).

Parallels have been drawn between spiritual and kundalini awakenings and psychopathologies such as bipolar disorder and schizophrenia (Lukoff, 1985; Oxman et al., 1988; Johnson and Friedman, 2008) both by psychiatrists (Menezes and Moreira-Almeida, 2010) and transpersonal psychologists (Lukoff, 1985; Lukoff et al., 1998; Grof and Grof, 2017), though attempts have been made to separate both sets of experiences. Recent studies observing the differences and similarities between spiritual emergency [using the Spiritual Emergency Scale (SES); Goretzki et al., 2009, 2014], and psychosis, have found spiritual emergency to diverge significantly from psychosis in alogia (Bronn and McIlwain, 2015; Storm and Goretzki, 2021), and in depression, anxiety, and stress (Bronn and McIlwain, 2015). Thus, while overlaps are considerable, spiritual and kundalini awakenings (including spiritual emergencies) are generally understood to not be indicative of psychopathology, even if they may be psychologically challenging at times (Goretzki et al., 2009, 2014; Bronn and McIlwain, 2015; St Arnaud and Cormier, 2017). A multicultural approach to understanding spiritual experiences and their effects culminated in addition of the *Religious or Spiritual Problem* diagnostic category (American Psychiatric Association, 1994, 2013) in the fourth edition of the Diagnostic and Statistical Manual. While spiritual and kundalini awakenings are no longer considered psychopathological by default (Johnson and Friedman, 2008; Menezes and Moreira-Almeida, 2010), the accurate diagnosis of experiences falling under the *Religious or Spiritual Problem* category remains challenging, partly because still too little is known about spiritual experiences and how these interact, interlink, or overlap with psychopathology, and partly due to a lack of spiritually-informed clinicians who have a bias towards the pathologisation of extreme anomalous experiences (Menezes and Moreira-Almeida, 2010; Parnas and Henriksen, 2016). As a result, the conventional psychiatric model is still overwhelmingly more likely to interpret potentially healing spiritual experiences as nothing more than mere psychopathology (St Arnaud and Cormier, 2017). This lack of understanding on behalf of clinicians remains problematic both for patients undergoing psychotic states with mystical features (which may be indicative of psychopathology), and patients undergoing non-pathological, though potentially distressing, spiritual or kundalini awakenings (which may be indicative of spiritual emergency) (Lukoff, 1985). An inappropriate treatment of either group may result in harm (Johnson and Friedman, 2008; Grof and Grof, 2017), and may trigger negative symptoms in positively perceived awakening experiences, or intensify the negative symptoms of spiritual emergency (Turner et al., 1995; Whitney, 1998). Either circumstance is likely to leave the individual in a state of trauma (Bragdon, 2006).

Spiritual experiences have been linked to temporal lobe epilepsy (TLE) (Naito and Matsui, 1988; Hansen and Brodtkorb, 2003; Özkara et al., 2004; Giovagnoli et al., 2006; Devinsky and Lai, 2008), with individuals experiencing auras of a spiritual nature, such as *autoscopy* (the experience of seeing oneself in the form of one's double, or through the lens of an out of body experience) (Devinsky et al., 1989), clairvoyance and telepathy (Özkara et al., 2004), déjà vu (Guedj et al., 2010), visual and auditory hallucinations of a religious or archetypal/symbolic nature, and the repetition of religious phrases (Hansen and Brodtkorb, 2003), during seizures (*ictally*) (Kanemoto, 1994; Ogata and Miyakawa, 1998), and after seizures (*postictally*) (Geschwind et al., 1980; Roberts and Guberman, 1989). Some of the common features of spiritual and kundalini awakenings have also been reported by TLE experiencers, including strong sensations of a cosmic, divine, or “God-like” presence or energy, and a sense of being connected with the infinite (oneness) (Zohar and Marshall, 2000; Hyde, 2004; Dolgoff-Kaspar et al., 2011). The partial seizure-like symptoms characteristic of TLE, namely temporal lobe *lability* (TLL), have therefore been used as a predictor measure for drug and non-drug induced altered states of consciousness (ASCs). Recent studies have indicated the links between TLL and mystical experiences occasioned by drug and non-drug induced ASCs (Luke et al., 2018, 2019). The potential links between TLL and spiritual and kundalini awakenings therefore warrants further exploration.

Personality trait absorption measures the depth to which one's attentional and experiential involvement occurs in relation to internal or external stimuli without effort or control (Tellegen and Atkinson, 1974), and has been used to measure proclivity for ASCs (Hunt, 2000; Luhrmann, 2017; Lifshitz et al., 2019). It has been found to be a good predictor of altered states produced by psychedelic substances (Haijen et al., 2018) such as psilocybin (Studerus et al., 2012; Studerus, 2013), LSD (Carhart-Harris et al., 2015; Terhune et al., 2016), MDMA (Hastings, 2006), ayahuasca (Bresnick and Levin, 2006), and ayahuasca's active ingredient, DMT (Timmermann et al., 2018). It is also a good predictor of mystical and quasi-mystical experiences produced endogenously in contexts such as the anechoic dark room (Luke et al., 2018), the whole-body perceptual deprivation tank (WBPD; Glicksohn and Ben-Soussan, 2020), and during guided “shamanic journey” visualisation (Rock, 2009). Traditionally, the trait has been associated with “fantasy proneness,” hypnotisability, imagery ability, openness to experiences (McCrae and Costa, 1983; Pekala et al., 1985; Roche and McConkey, 1990; Glisky et al., 1991), alterations in body image, time-space perception, and meaning (Pekala et al., 1985; Kumar and Pekala, 1988), higher emotional sensitivity and emotional brain processing (McCrae and Costa, 1983; Benning et al., 2015), stronger empathy (Wickramasekera and Szlyk, 2003; Wickramasekera, 2007), stronger flow states (Marty-Dugas and Smilek, 2019), intellectual curiosity (McCrae and Costa, 1983), more pronounced creativity and engagement in the arts (Wild et al., 1995; Manmiller et al., 2005), positive emotional responses to music (Rhodes et al., 1988), more pronounced experiences of synaesthesia (Rader and Tellegen, 1987; Glicksohn et al., 1999; Chun and Hupé, 2016), and an attachment to nature and other forms of life (Kaplan, 1995; Brown and Katcher, 1997), relative to the general population. The trait of absorption has also been associated with experiences of dissociation (Carleton et al., 2010), hallucinations (Glicksohn and Barrett, 2003; Glicksohn, 2004; Perona-Garcelán et al., 2013, 2016), and paranormal beliefs or experiences (Glicksohn, 1990, 2004; Spanos et al., 1993; Glicksohn and Barrett, 2003; French et al., 2008; Parra, 2008; Zingrone et al., 2009; Luhrmann et al., 2010, 2021; Gray and Gallo, 2016; Parra and Gimenez Amarilla, 2016), such as hearing voices or feeling spiritual presences (Granqvist et al., 2005; Luhrmann et al., 2010, 2021), and feelings of self-transcendence (Cardeña and Terhune, 2014). Absorption is associated with porosity, the degree to which one identifies the outside world and its events as permeable with the inner world (Luhrmann et al., 2021); and transliminality, the subconscious tendency for internal or external material to “cross the threshold of consciousness” (Lange et al., 2000; Houran et al., 2003). Deeper states of absorption can be cultivated through ritual, including communal repetitive behaviours such as chanting or drumming, meditation (Bronkhorst, 2016), prayer (Luhrmann et al., 2010), and the disruption of homeostatic balance, including through the ingestion of certain psychotropic substances (Bronkhorst, 2016).

Studies investigating the phenomenology of mystical experiences produced by certain drugs such as strong psychedelic compounds psilocybin and DMT, their therapeutic potential, and long-term impacts on well-being (e.g., Carhart-Harris et al., 2012a,b, 2016; Garcia-Romeu et al., 2014; Griffiths et al., 2016; Ross et al., 2016; Noorani et al., 2018), suggest close similarities with spontaneously occurring spiritual and kundalini awakenings, however, little research has been conducted on the latter. Furthermore, comparisons between the phenomenological distributions of various measured drug and non-drug induced ASCs have revealed strong phenomenological similarities between both sets of ASCs, as well as similar predictors of the experiences (Luke et al., 2019), including TLL and trait absorption, suggesting the potential for some of these same measures to be applied to study spiritual and kundalini awakenings.

Psychological research on spiritual and kundalini awakenings is still in its infancy and has tended not to focus on experiences of a sudden, spontaneous nature. Studies investigating the impact of mystical experiences similar to spiritual and kundalini awakenings, on well-being, have recognised the predominantly positive, healing effects of these experiences, but have also acknowledged some of the more challenging aspects brought on both by their disruptive nature and by their typically biased clinical interpretations. The subtle phenomenological differences between spiritual awakenings and kundalini awakenings have seldom been explored, despite a greater number of studies addressing the strong physical nature of kundalini awakenings, compared to spiritual awakenings. The interchangeable use of these terms could be problematic in the interpretation of these experiences and of their outcomes, especially as stronger physical experiences may equate to more challenging outcomes. Neuroscientific and psychological research has explored some of the phenomenological and neurobiological underpinnings of drug and non-drug induced ASCs, and has explored the links between the spiritual characteristics of ASCs and the symptoms of TLE and of trait absorption. However, Spontaneous Spiritual and Kundalini Awakenings (SSA/SKAs) have not yet been mapped within the ASC framework, nor have the typical predictors used to study ASCs (TLL and absorption) been analysed as effective predictors of SSA/SKAs.

This paper will aim to address some of the gaps in the literature by exploring the general characteristics of SSA/SKAs, their implications on well-being, how they compare to other measured ASCs, their relationships with TLL and absorption, and the potential phenomenological differences between them. Given the prominence of anecdotal recounts of physical and energetic experiences preceding challenging kundalini experiences, the authors hypothesise not only that Spontaneous Kundalini Awakenings (SKAs) are more physical than Spontaneous Spiritual Awakenings (SSAs), but that they are also more likely to produce negative experiences. Spontaneous Spiritual and Kundalini Awakenings will subsequently be mapped within the ASC framework by comparing their phenomenological distribution against a backdrop of non-drug and drug-induced ASCs. Analysis will then be conducted to test the hypothesis that TLL and trait absorption predict the intensity of the SSA/SKA ASC, following similar protocol for the study of induced ASCs. Further analysis will be conducted to understand how the population distribution of the SSA/SKA sample compares with the distribution of the published “normal” samples for TLL and absorption. The short and long-term well-being impacts of these experiences will be explored.

## Methodology

### Design

A quantitative, mixed within and between-participants self-report survey design was adopted. There were four outcome variables: intensity of SSA/SKA experiences, measured with the Nondual Embodiment Thematic Inventory (NETI), the Kundalini Awakening Scale (KAS), and the 30-item Mystical Experience Questionnaire (MEQ30); and depth of ASC, measured with the 11-Dimensional Altered States of Consciousness Rating Scale (11D-ASC). There were two predictor variables: TLL, measured with the Iowa Interview for Partial Seizure-like Symptoms (IIPSS); and trait absorption, measured with the Modified Tellegen Absorption Scale (MODTAS). Statistical analysis was performed using IBM SPSS Statistics.

### Participants

Participants were recruited to take part in the survey through social media. The experience was described as follows: “Have you ever had a strong experience of a profound spiritual nature in which you suddenly and non-intentionally felt in contact, or communion with something that is considered to be an ultimate reality, ‘God’, or the divine? Have you ever felt that your ego suddenly transcended beyond an ordinary personal identity in space and time, and that you became ‘one’ with the universe?” All participants were aged over 18 and must have experienced SSA/SKA at least once in their lives. Participants who had experienced SSA/SKA under the influence of psychoactive drugs were excluded. Participation was voluntary.

A total of 153 respondents completed the survey but one was excluded as their experience was mediated by psychedelic substance use, so only data from 152 participants were analysed. Of these, 55.3% identified as female, 42.8% as male, 1.3% as non-binary, and 0.7% as other. Ages ranged from 18 to 69 years (*N* = 144, *M* = 41.09 years, *SD* = 11.60 years). Reported ethnicity was 74.3% white, 11.8% mixed/multiple ethnic groups, 9.2% Asian or Asian British, 0.7 black/African/Caribbean black British, and 3.9% other. The participants' reported religious/spiritual belief was 63.2% spiritual but not religious, 5.3% Christian, 5.3% Hindu, 2.6% Agnostic, 2% Buddhist, 2% Muslim, 1.3% Atheist, 0.7% Jewish, and 17.8% other, of which 2% identified as Omnist. Seventy-three percent of participants reported having experienced more than one SSA and/or SKA in their lives, vs. 27% having experienced it just once.

### Ethical Considerations

Full ethical approval was granted for the study by the University of Greenwich Departmental Research Ethics Committee, which conformed both to British Psychological Society (BPS) guidelines and to GDPR standards. The study was not expected to involve any obvious risks to physical or mental health and helplines were provided in this unlikely event. Participants were fully briefed before consenting and debriefed upon submitting. Participants were advised that they could omit to answering any question they may not have wished to answer, and of their freedom to withdraw from the study at any time. Participants were also advised of their right to withdraw their data from the study at any point until data processing. Participants were made aware that data would be kept on a password-secured computer, and that any publication would ensure strict anonymity.

### Measures

#### The Nondual Embodiment Thematic Inventory

The Nondual Embodiment Thematic Inventory (NETI; Butlein, 2005), is a 20-item unitary scale, developed to measure the qualities of spiritual awakening and nondual experiences. Scoring is on a 5-point Likert-type scale, from 1 (never) to 5 (all of the time). Items 4, 8, 14, and 16 were reversed scored. Total scores range from 20 to 100. Cronbach's alpha for the scale is 0.91, indicating high internal consistency since α > 0.70, the accepted cut-off point for high internal consistency as indicated by Mills et al. (2018). Cronbach's alpha indicates good internal consistency across the whole scale for the SSA/SKA sample (*N* = 152), since α = 0.87, in line with previously published figures. Construct validity was also observed (Mills et al., 2018).

#### The Kundalini Awakening Scale

The Kundalini Awakening Scale (KAS; Sanches and Daniels, 2008), is a 76-item scale, developed to measure the effects of kundalini awakening experiences. The measure is composed of five subscales: changes (15 items); involuntary positionings (3 items); physical symptoms (20 items); negative experiences (12 items); and positive experiences (9 items). Scoring is on a 7-point Likert-type scale, from 1 (strongly disagree) to 7 (strongly agree). Total scores range from 76 to 532. Reported Cronbach's alpha for the whole scale is 0.98, indicating high internal consistency (Sanches and Daniels, 2008). Cronbach's alpha scores indicate good internal consistency across all subscales for the SSA/SKA sample (*N* = 152) (changes: α = 0.86; involuntary positionings: α = 0.80; physical symptoms: α = 0.92; negative symptoms: α = 0.86; positive symptoms: α = 0.80), in line with previously published figures. Figures are unavailable for construct validity.

#### The 30-Item Mystical Experience Questionnaire

The 30-Item Mystical Experience Questionnaire (MEQ30; MacLean et al., 2012), is a 30-item scale, developed to measure the intensity of mystical experiences. The measure is composed of four subscales: mystical (15 items); positive mood (6 items); transcendence of time and space (6 items); ineffability (3 items). Scoring is on a 6-point Likert-type scale, from 0 (none) to 5 (extreme). Total scores range from 0 to 150, with a cut-off indicating “complete mystical experience” when total scores on each four subscales ≥60%. High internal consistency is reported for the whole scale (α = 0.93), and subscales (α = 0.93; α = 0.83; α = 0.81; α = 0.80, respectively) (MacLean et al., 2012). Cronbach's alpha scores indicate higher internal consistency across all subscales for the SSA/SKA sample (*N* = 152) (mystical: α = 0.94; positive mood: α = 0.86; transcendence of time and space: α = 0.88; ineffability: α = 0.85), in line with previously published figures. The measure has been validated for psychedelic-induced experiences and construct validity was also observed (MacLean et al., 2012).

#### The 11-Dimensional Altered States of Consciousness Rating Scale

The 11-Dimensional Altered States of Consciousness Rating Scale (11D-ASC; Studerus et al., 2010), is a 42-item scale, developed from the 94-item 5-Dimensional Altered States of Consciousness Rating Scale (5D-ASC) (Dittrich, 1998) to measure the intensity of ASCs. The measure is composed of 11 subscales: experience of unity (5 items); spiritual experience (3 items); blissful state (3 items); insightfulness (3 items); disembodiment (3 items); impaired control and cognition (7 items); anxiety (6 items); complex imagery (3 items); elementary imagery (3 items); audio-visual synaesthesia (3 items); changed meaning of percepts (3 items). The continuous rating scale runs from 0 (no, not more than usually) to 10 (yes, much more than usually), and item scores from the SSA/SKA sample were standardised to 0 to 100 (percentage), giving total scores ranging from 0 to 4,200. High internal consistency can be observed across each subscale (α = 0.88; α = 0.77; α = 0.82; α = 0.73; α = 0.82; α = 0.85; α = 0.89; α = 0.80; α = 0.84; α = 0.91; α = 0.79, respectively) (Studerus et al., 2010). Cronbach's alpha indicates good internal consistency scores across most subscales in the SSA/SKA sample (*N* = 152), apart from three subscales, which showed somewhat lower internal consistency than previously published figures [experience of unity: α = 0.89; spiritual experience: α = 0.57 (3 items: α < 0.70); blissful state: α = 0.86; insightfulness: α = 0.66 (3 items: α < 0.70); disembodiment: α = 0.84; impaired control and cognition: α = 0.82; anxiety: α = 0.87; complex imagery: α = 0.55 (3 items: α < 0.70); elementary imagery: α = 0.88; audio-visual synaesthesia: α = 0.87; changed meaning of percepts: α = 0.76]. Figures are unavailable for construct validity.

#### The Modified Tellegen Absorption Scale

The Modified Tellegen Absorption Scale (MODTAS; Jamieson, 2005), is a 34-item scale, developed from the Tellegen Absorption Scale (TAS) (Tellegen and Atkinson, 1974) to measure states of absorption, the tendency to have one's attention absorbed in a task or stimulus. This measure has been shown to predict depth of non-ordinary states of consciousness. The measure is composed of five subscales: synaesthesia (4 items); ASC (4 items); aesthetic involvement in nature (5 items); imaginative involvement (9 items); ESP (3 items). Scoring is on a 5-point Likert-type scale, from 0 (never) to 4 (very often). Total scores range from 0 to 136. Recent studies indicate good internal consistency [e.g., Cronbach's α 0.96 (Terhune et al., 2016) and 0.94–0.95 (Andrei et al., 2016)]. Cronbach's alpha scores indicate good internal consistency across all subscales for the SSA/SKA sample (*N* = 152) [synaesthesia: α = 0.83; ASC: α = 0.69 (4 items: α < 0.70); aesthetic involvement in nature: α = 0.78; imaginative involvement: α = 0.81; ESP: α = 0.77]. Figures are unavailable for construct validity.

#### The Iowa Interview for Partial Seizure-Like Symptoms

The Iowa Interview for Partial Seizure-Like Symptoms (IIPSS; Roberts, 1999), is a 40-item scale, developed to measure different cognitive, affective, and sensory symptoms that may be indicative of epileptiform disturbances of the temporal lobe. The scale has been used to help identify partial seizure-like symptoms, characteristic of TLE. The measure is composed of four subscales: sensory; cognitive; affective; nocturnal, but has been used as a unitary scale for the purpose of this study. Scoring is on a 7-point Likert-type scale, from 0 (never, or not in the past year) to 6 (more than once a day), besides item 31, which is a dichotomous yes/no question, and is thus scored as either 0 or 6. Total scores range from 0 to 240. No published figures are available for internal consistency or construct validity.

Additional questions were asked to understand whether SSA/SKA experiences were considered predominantly positive or negative in the short and long-terms.

### Procedure

The survey was dispersed on social media (e.g., Facebook), where participants were recruited and directed to a survey link. All data was collected using Qualtrics survey software. Upon consenting, participants were invited to take part in the survey, which took approximately 45 min to complete. For the sake of consistency and to measure most powerful awakening experience, participants were asked to recall and focus on their one most powerful SSA or SKA for the rest of the survey, even if they had experienced multiple SSAs and/or SKAs during the course of their lives. Participants were asked to report their choice of either an SSA or SKA according to their subjective interpretation. The item, which was positioned in the first part of the survey (demographics section), read: “For the remainder of the survey, please consider only your most powerful SSA or SKA, if you've experienced more than one. Please specify how you would classify the experience you will be referring to” (as either SSA or SKA). Upon completion, participants were debriefed.

## Results

Of all participants, 60.5% chose to recall their most powerful SSA and 39.5% chose to recall their most powerful SKA. The age of onset for participants' most powerful SSA/SKA ranged from “10 years or less” to 67 years (*N* = 148, *M* = 32.78 years, *SD* = 10.60 years). Of all participants, 21.1% responded that the peak of their experience lasted minutes, 10.5% hours, 7.9% days, 9.2% weeks, 14.5% months, 7.9% years, and 28.9% responded that the peak of their experience was still ongoing.

Responding to the multiple choice question: “Considering your most powerful SSA or SKA, do you think there were any significant factors that led you to have this experience,” 52% reported psychological turmoil/trauma (e.g., stress, depression, loss, bereavement, combat, addiction), 47.4% meditation practice, 31.6% spiritual literature, 21.7% contact with nature, 21.7% past use of psychedelics or entheogens, 18.4% yoga practice, 13.2% near-death experience, 11.8% breathwork (e.g., Wim Hof method, Holotropic Breathwork, pranayama), 11.2% sacred sexual intimacy, 9.9% fasting, 9.2% no discernible trigger that they were aware of, 9.2% physical injury, 8.6% lucid dreaming, 8.6% sleep deprivation, 7.9% athletic activity, and 39.9% “other” factors.

For regularly practised activities *before* the onset of participants' most powerful SSA/SKA, contact with nature was the most reported activity (68.4%) and Kundalini Yoga was the least reported activity (11.2%). For regularly practised activities *after* participants' most powerful SSA/SKA, meditation was the most reported activity (79.6%) and “other yoga” was the least reported activity (21.1%) (Table 1). An average increase was observed across all activities post-SSA/SKA, with the exception of the use of psychedelics and entheogens, where a small decrease was observed (Table 1).

**Table 1:** Reported regularly practised activities before and after the participants' most powerful SSA/SKA.

**Regularly practised** **activities**	**Before** **(% of sample)**	**After** **(% of sample)**	**Difference** **(% of sample)**
Contact with nature	68.4	74.3	5.9
Spiritual literature	58.6	75	16.4
Mindfulness	55.9	73.7	17.8
Meditation	54.6	79.6	25
Lucid dreaming	37.5	49.3	11.8
Athletic activity	34.9	40.1	5.2
Psychedelics/ Entheogens	30.3	26.3	−4
Breathwork	28.3	53.9	25.6
Fasting	27	31.6	4.6
Sleep deprivation	23.7	26.3	2.6
Other yoga	22.4	29.6	7.2
Hatha yoga	19.1	24.3	5.2
Sacred sexual intimacy	18.4	34.9	16.5
Other	15.8	21.1	5.3
Tantra	12.5	21.7	9.2
Kundalini yoga	11.2	24.3	13.2

Of all participants, 83.6% reported that “practising these activities following the onset of their most powerful SSA/SKA” helped them manage the experience, 6.6% reported that they did not affect the management of their experience, 2.6% reported that they worsened their experience, 6.6% reported that the question did not apply, and 0.7% chose not to disclose their answer.

### SSA/SKA IIPSS Scores Compared to Published “Normal” Population

**Figure 1:**
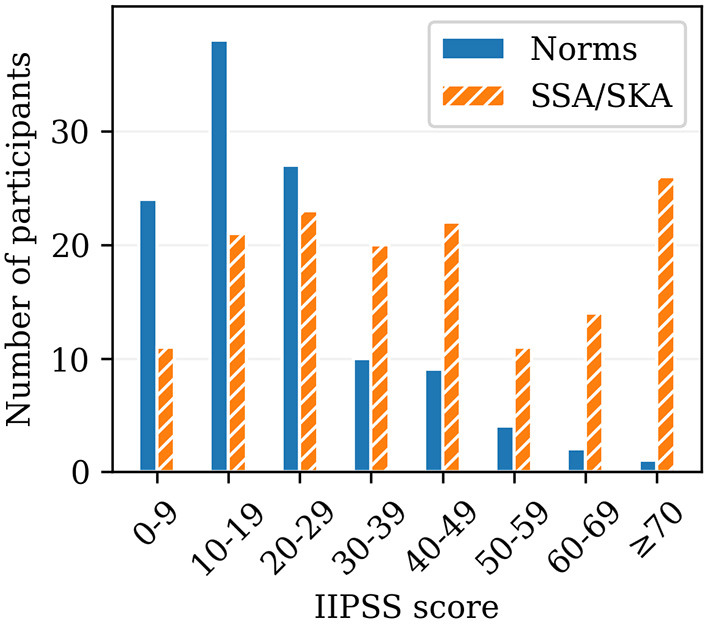
Histogram indicating score distribution of the Iowa Interview for Partial Seizure-like Symptoms (IIPSS; Roberts, 1999) for the previously published “normal” population (Norms; *N* = 115; Roberts, 1999) and for the participants of this study (SSA/SKA; *N* = 148).

Spontaneous Spiritual and Kundalini Awakenings IIPSS scores measuring TLL symptoms (*N* = 148) were compared to scores from the published “normal” population (*N* = 115) (Roberts, 1999). Figure 1 shows the histogram of both score distributions, which reveals that mean scores from the SSA/SKA sample were higher than those from the “normal” population, the largest differences being present in the high score range. A significantly large proportion of our participants scored very high (>50), whereas very few people scored as highly in the “normal” population (Figure 1), suggesting more pronounced TLL traits in our participants [χ^2^_(1)_ = 40.16, *p* < 0.001].

### SSA/SKA MODTAS Scores Compared to Published “Normal” Population

**Figure 2:**
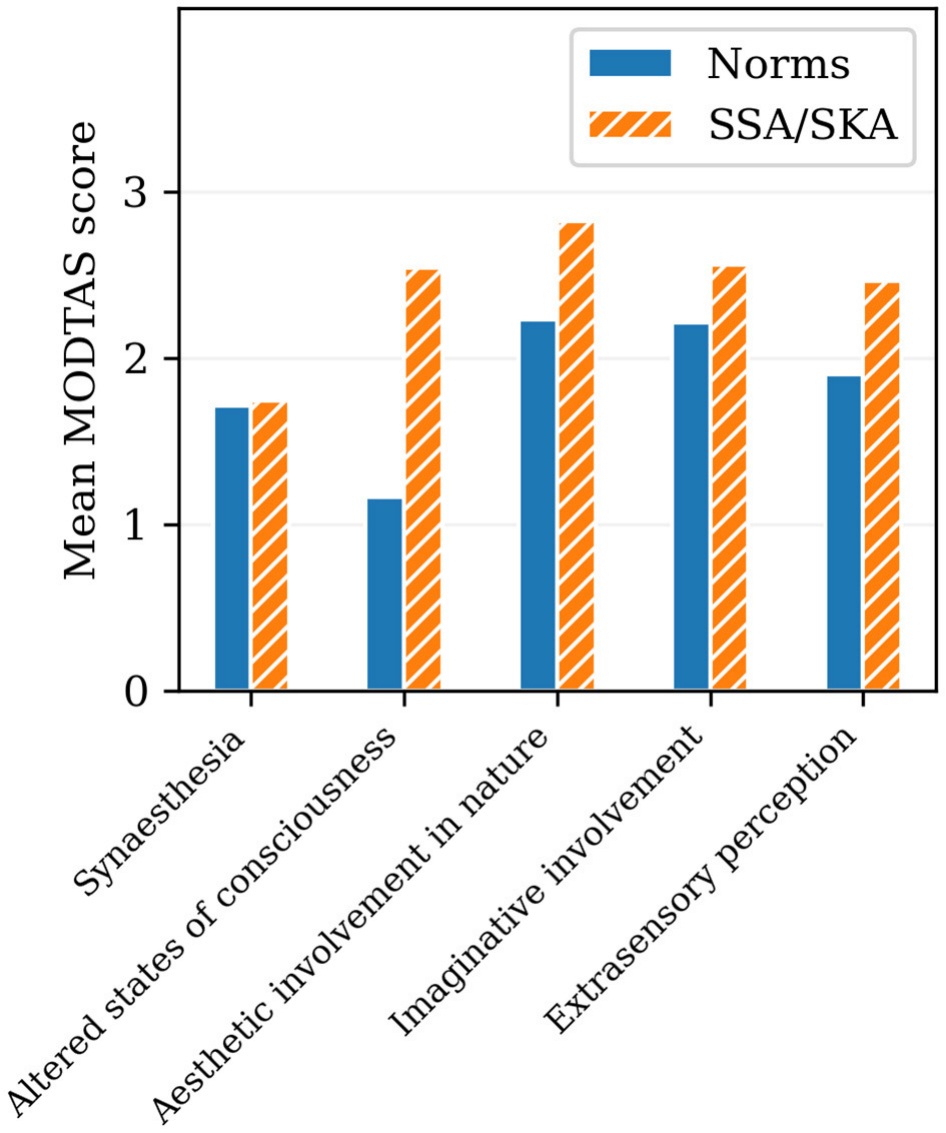
Bar chart comparing the mean subscale scores of the Modified Tellegen Absorption Scale (MODTAS; Jamieson, 2005) for the published “normal” population (Norms; *N* = 352; Jamieson, 2005) and for the participants of this study (SSA/SKA; *N* = 152).

Spontaneous Spiritual and Kundalini Awakenings MODTAS mean subscale scores measuring trait absorption (*N* = 152) were compared to mean subscale scores from the published “normal” population (*N* = 352) (Jamieson, 2005). Scores from both groups were relatively similarly distributed, with the exception of the ASC subscale in the SSA/SKA group, where the mean score was more than double that of the “normal” population (SSA/SKA: *M* = 2.54; Norms: *M* = 1.16) (Figure 2). Scores for items 8 “I think I really know what some people mean when they talk about mystical experiences” and 9 “I can step outside my usual self and experience an entirely different state of being,” belonging to the ASC subscale, were more than double those of the “normal” population.

### The Characteristics of SSA/SKA Experiences

#### NETI Scores

Fifteen out of 20 items scored higher than 3, indicating general agreement with their corresponding statements. The highest scoring item was “An interest in clearly seeing the reality or truth about myself, the world, and others, rather than in feeling a particular way” (*M* = 4.51, *SD* = 0.72), and the lowest scoring item was “A sense of fear or anxiety that inhibits my actions” (*M* = 2.26, *SD* = 1.06) (Table 2). The four items relating to negative experience (4, 8, 14, and 16) scored the lowest (when reversed scoring was not applied). Mean scores for each item are reported in Table 2.

**Table 2:** Highest to lowest mean scores of the 20-item Nondual Embodiment Thematic Inventory (NETI; Butlein, 2005), relating to the phenomenological features of SSA/SKA (*N* = 152).

**Items**	***M***	***SD***
An interest in clearly seeing the reality or truth about myself, the world, and others, rather than in feeling a particular way.	4.51	0.72
Feelings of gratitude and/or open curiosity about all experiences.	4.35	0.85
Deep love and appreciation for everyone and everything I encountered in life.	4.23	0.84
Understanding that there is ultimately no separation between what I call my “self” and the whole of existence.	4.22	0.97
A sense of the flawlessness and beauty of everything and everyone, just as they are.	4.20	0.92
A sense of immense freedom and possibility in my moment-to-moment experience.	4.13	0.97
Conscious awareness of my non-separation from (essential oneness with) a transcendent reality, source, higher power, spirit, god, etc.	4.13	1.05
A feeling of profound aliveness and vitality.	4.11	0.86
Accepting (not struggling with) whatever experience I may have been having.	3.97	0.99
An inner contentment that was not contingent or dependent upon circumstances, objects, or the actions of other people.	3.93	1.12
An unwavering awareness of a stillness/quietness, even in the midst of movement and noise.	3.87	0.98
Feeling deeply at ease, wherever I was or whatever situation or circumstance I may have found myself in.	3.87	1.05
Acting without assuming a role or identity based on my own or others' expectations.	3.78	1.07
Acting without a desire to change anybody or anything.	3.70	1.13
Not being personally invested in, or attached to, my own ideas, and concepts.	3.48	1.14
A desire to be understood by others.	2.85	1.21
A sense that my actions in life were motivated by fear or mistrust.	2.74	1.22
A sense that I was protecting or defending a self-image or concept I held about myself.	2.54	1.22
Concern or discomfort about either the past or future.	2.38	1.11
A sense of fear or anxiety that inhibited my actions.	2.26	1.06

#### KAS Scores

The highest scoring subscale was changes (*M* = 5.80, *SD* = 0.84), followed by positive experiences (*M* = 5.48, *SD* = 1.07), physical symptoms (*M* = 4.20, *SD* = 1.30), negative experiences (*M* = 4.11, *SD* = 1.32), and involuntary positionings (*M* = 4.01, *SD* = 1.87). Results for the ten highest and ten lowest scoring items are reported in Tables 3A,B, respectively. The detailed results for all 76 items are provided in the Supplementary Material.

**Table 3:** Top 10 **(A)** highest and **(B)** lowest scoring items of the 76-item Kundalini Awakening Scale (KAS; Sanches and Daniels, 2008).

**Items**	***M***	***SD***
**(A) Highest Scoring Items**		
I experienced an elevation of my consciousness.	6.67	0.97
I felt that my mind started to function differently.	6.61	0.86
I felt in touch with something measureless.	6.57	0.90
I experienced an expansion of my being.	6.53	1.06
I had experiences of elevation and bliss.	6.47	1.16
My consciousness has become wider than it was before my experience.	6.47	1.07
I'm aware of some deep changes in my personality since my experience.	6.45	1.07
I've felt humbled by the contact with a transcendent reality.	6.36	1.13
I had an experience of union with some divine force or energy.	6.34	1.41
I feel that I have developed a new channel of communication within me, since my experience.	6.27	1.26
**(B) Lowest Scoring Items**		
I experienced seeing people or objects that weren't materially present and this made me feel scared and frightened.	3.07	2.10
I experienced an unusual cold only in a specific part of my body.	3.14	1.83
I experienced an unusual cold in my body moving from place to place.	3.22	1.97
I experienced a temporary incapability to read.	3.36	2.17
I experienced a halo in my head which increased after a spell of prolonged concentration.	3.45	2.12
I felt very depressed.	3.54	2.31
I experienced an odd functioning of my reproductive system.	3.58	2.13
I experienced an odd functioning of my excretory system without an apparent physical cause.	3.65	2.23
I sensed unusual cold inside my body, or on my skin.	3.68	2.27
I experienced a shining halo emanating from my head.	3.76	2.16

#### MEQ30 Scores

Ineffability (*M* = 4.29, *SD* = 0.95) was found to be the highest scoring subscale, followed by positive mood (*M* = 4.17, *SD* = 0.87), mystical (*M* = 4.10, *SD* = 0.94), and transcendence of time and space (*M* = 3.44, *SD* = 1.23). Only one item's mean score, “Loss of usual awareness of where you were” (item 11), fell beneath 3 (*M* = 2.76, *SD* = 1.72), indicating general disagreement with the statement. A full breakdown of MEQ30 item scores is listed in the Supplementary Material. The number of participants having experienced a complete mystical experience was then calculated by selecting total subscale means equal or greater than the cut-off score of 3 (60%), the threshold for a complete mystical experience (MacLean et al., 2012). Of all participants, 63.2% met the criteria for a complete mystical experience.

### Mental Well-Being Implications of SSA/SKAs

When participants were asked whether they felt that their experience had been predominantly positive or negative in the *short-term*, 90.8% responded that the experience was positive, and 9.2% responded that it was negative. However, when participants were asked whether they felt that their experience had been predominantly positive or negative in the *long-term*, 98% responded that the experience was positive, and only 2% responded that it was negative, implying that SSA/SKAs were more likely to be perceived as positive in the long-term even following negative short-term experiences, but that both short and long-term effects were predominantly positive.

### SSA vs. SKA

Our first hypothesis was tested by comparing the reported experiences of SSAs to those of SKAs using the KAS, MEQ30, and 11D-ASC scales. Independent samples *t*-tests with a Bonferroni correction were conducted to compare the mean subscale scores between the groups. A statistically significant difference was observed between the groups in the KAS subscales of involuntary positionings [*t*_(150)_ = 4.21, *p* < 0.001, mean difference = 1.24] and physical symptoms [*t*_(150)_ = 4.50, *p* < 0.001, mean difference = 0.91]. Spontaneous Kundalini Awakenings scored higher for both. A significance level of α = 0.05/5 = 0.01 was used. No statistically significant difference was observed between the groups in the MEQ30 and 11D-ASC scales.

Participants' responses to the “significant factors” questionnaire item were compared between the SSA and SKA groups using a chi-square test of independence. Results show a statistically significant association between the SKA group and prior yoga practice, χ^2^_(1, N=152)_ = 8.84, *p* = 0.003. Results also show a significant association between the SSA group and no discernible trigger χ^2^_(1, N=152)_ = 4.10, *p* = 0.04.

No significant differences were found between the SSA and SKA groups in duration of experience.

### SSA/SKA Altered States vs. Non-drug Altered States

Mean scores ranged from 28.67 (*SD* = 26.85) for anxiety to 78.77 (*SD* = 26.45) for blissful state.

The SSA/SKA 11D-ASC mean subscale scores were visually compared to those of non-drug altered states, specifically to altered states produced in an anechoic darkroom chamber, a floatation tank, through Holotropic Breathwork (Luke et al., 2019), and a ganzfeld environment (classic white noise) (Schmidt and Prein, 2019). Spontaneous Spiritual and Kundalini Awakenings appear similar in their relative phenomenological distribution (though not in magnitude) compared to all measured non-drug altered states.

**Figure 3:**
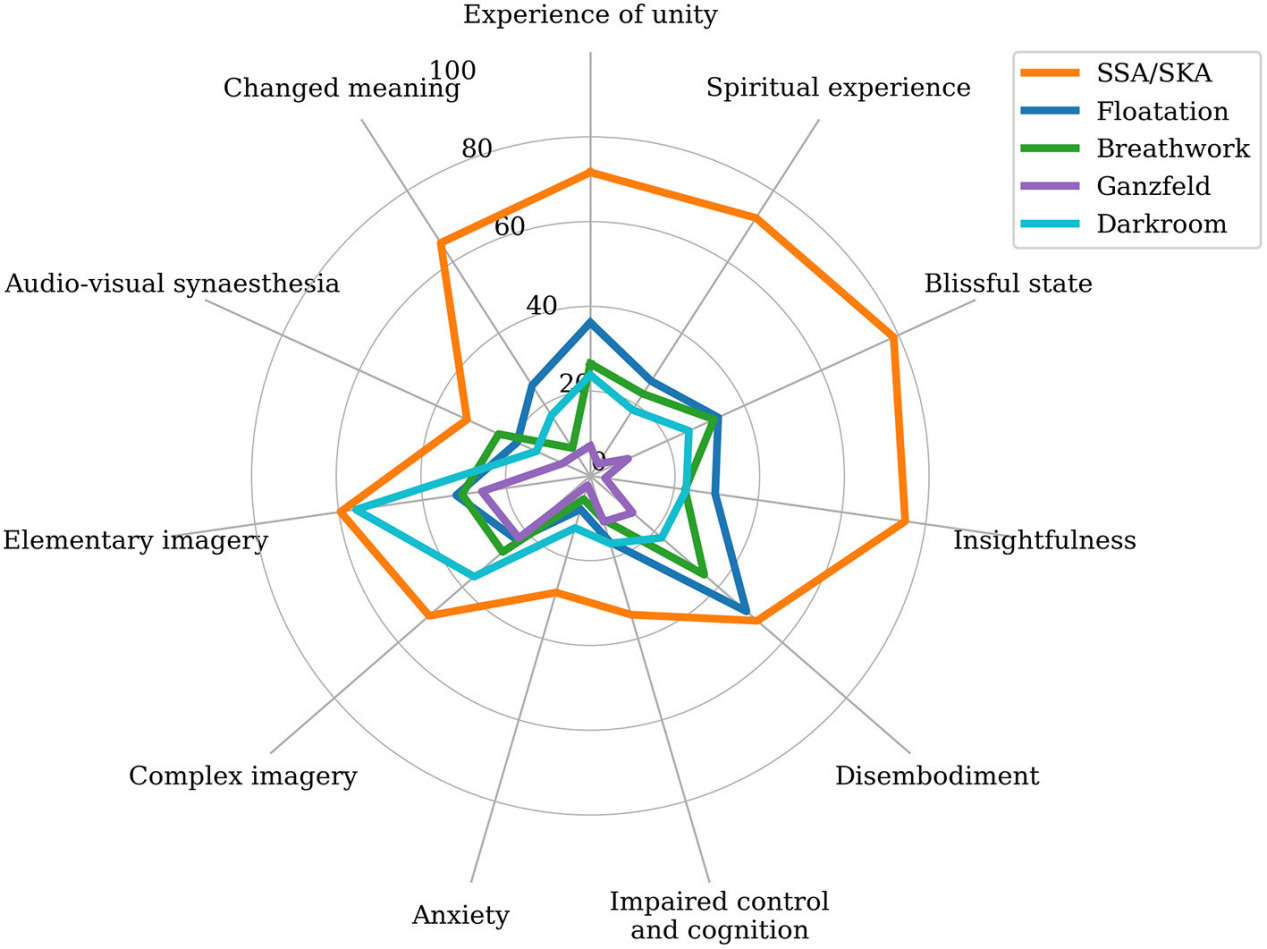
Radar chart comparing the mean subscale scores of the 11-Dimensional Altered States of Consciousness Rating Scale (11D-ASC; Studerus et al., 2010) for the participants of this study (SSA/SKA; *N* = 152) and for non-drug induced altered states of consciousness (ASCs) produced by the floatation tank (Floatation; 60 min; *N* = 27; Luke et al., 2019); Holotropic Breathwork (Breathwork; 60 min; *N* = 23; Luke et al., 2019); anechoic darkroom (Darkroom; 120 min; *N* = 46; Luke et al., 2018); and ganzfeld environment (Ganzfeld; 25 min; *N* = 22; Schmidt and Prein, 2019).

Figure 3 reveals considerably higher scores across all 11 dimensions for SSA/SKA ASCs compared to all other measured non-drug ASCs, with the exception of simple imagery (*M* = 59.61, *SD* = 33.72) compared with the darkroom ASC (*M* = 55.90), and disembodiment (*M* = 52.17, *SD* = 34.33) compared with the floatation tank ASC (*M* = 48.80), where observed scores are not significantly higher. This suggests that whilst SSA/SKAs and other measured non-drug ASCs are similar in their phenomenological distributions, recalled SSA/SKAs were generally considerably stronger than other individual ASC inductions across most dimensions.

### SSA/SKA Altered States vs. Drug Altered States

**Figure 4:**
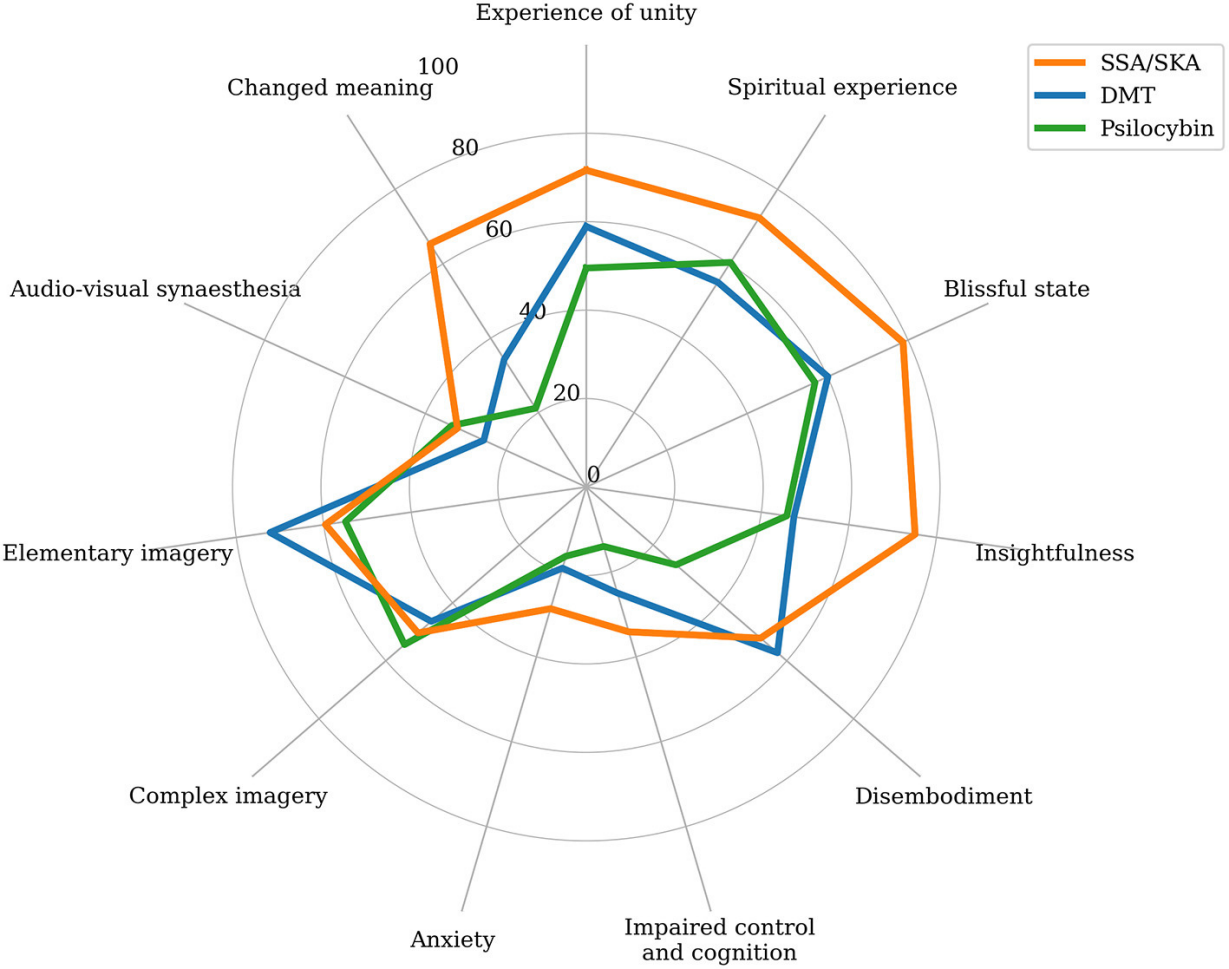
Radar chart comparing the mean subscale scores of the 11-Dimensional Altered States of Consciousness Rating Scale (11D-ASC; Studerus et al., 2010) for the participants of this study (SSA/SKA; *N* = 152) and for drug induced altered states of consciousness (ASCs) produced by high dose psilocybin (Psilocybin; 30 mg/70 kg; *N* = 20; Carbonaro et al., 2018); and high dose *N,N-*dimethyltryptamine (DMT; 40–75 mg; *N* = 35; Luke, 2019).

The SSA/SKA 11D-ASC mean subscale scores were visually compared to those of drug-induced ASCs, specifically: LSD 200 mcg (*N* = 16) (Schmid et al., 2015), MDMA 125 mg (*N* = 16) (Hysek et al., 2013), psilocybin 30 mg/70 kg (*N* = 20) (Carbonaro et al., 2018), dextromethorphan 400 mg/70 kg (*N* = 20) (Carbonaro et al., 2018), DMT 40–75 mg (*N* = 35) (Luke, 2019), and cannabis (*N* = 111) (Luke et al., 2019). Particularly striking were the similarities observed between the phenomenological distributions of SSA/SKAs and psilocybin, and SSA/SKAs and DMT (Figure 4), although mean SSA/SKA subscale scores are higher than both psilocybin and DMT in changed meaning, unity experience, spiritual experience, blissful state, insightfulness, anxiety, and impaired cognition; higher than DMT in synaesthesia and complex imagery; and higher than psilocybin in simple imagery.

**Figure 5:**
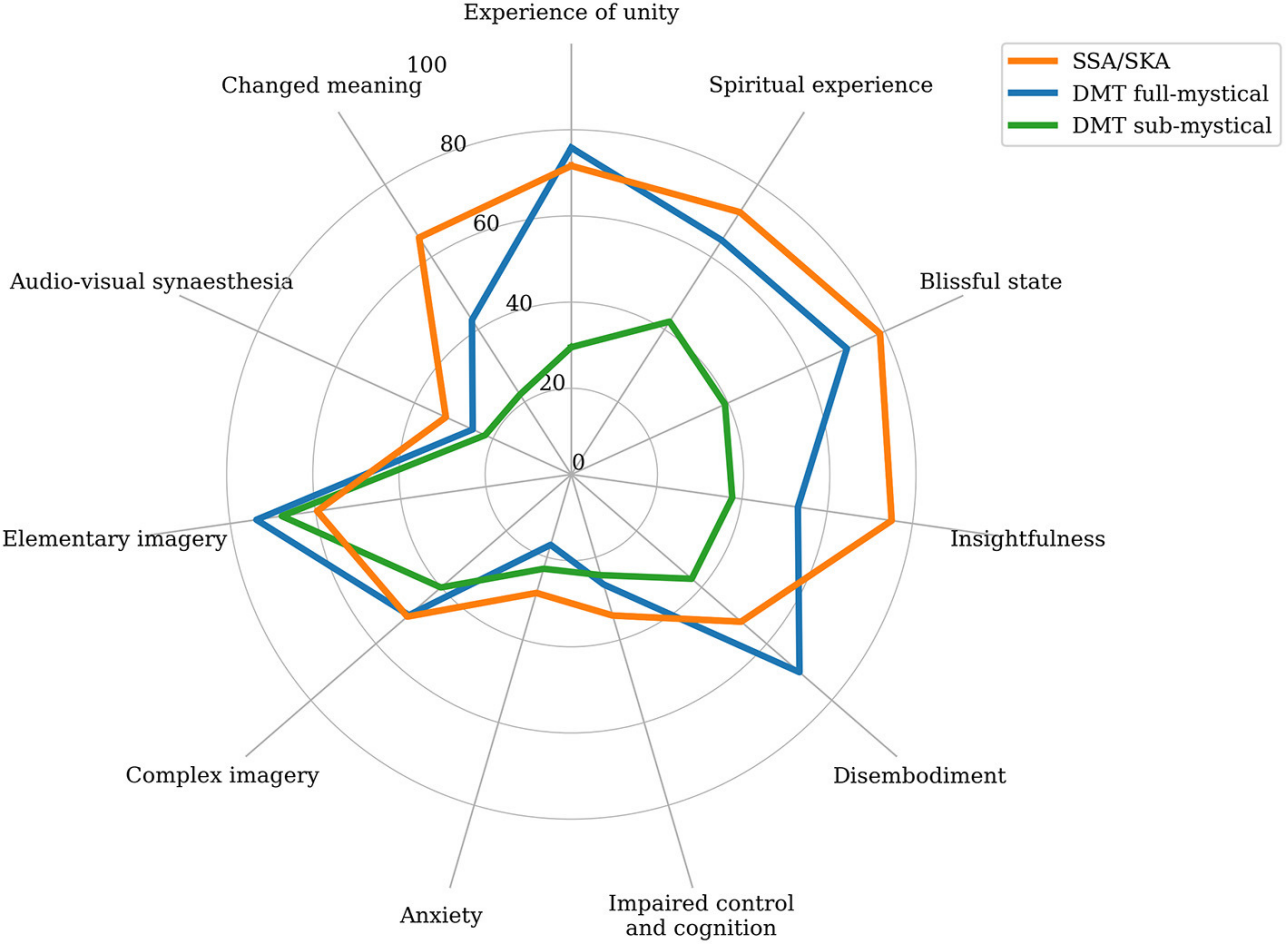
Radar chart comparing the mean subscale scores of the 11-Dimensional Altered States of Consciousness Rating Scale (11D-ASC; Studerus et al., 2010) for the participants of this study (SSA/SKA; *N* = 152); high dose *N,N-*dimethyltryptamine complete mystical experience (DMT full-mystical; 40–75 mg; *N* = 21; Luke, 2019); and high dose *N,N-*dimethyltryptamine sub-mystical experience (DMT sub-mystical; 40–75 mg; *N* = 14; Luke, 2019), as defined by the 30-item Mystical Experience Questionnaire (MEQ30; MacLean et al., 2012).

Spontaneous Spiritual and Kundalini Awakenings were visually compared to DMT sub-mystical experiences (40–75 mg; *N* = 14; Luke, 2019), and DMT complete mystical experiences (40–75 mg; *N* = 21; Luke, 2019) (Figure 5). Strongest similarities were observed between SSA/SKAs and DMT complete mystical experiences, where SSA/SKAs scored higher in spiritual experience, blissful state, insightfulness, impaired cognition, anxiety, synaesthesia, complex imagery, and changed meaning; and DMT complete mystical experiences scored higher than SSA/SKAs in simple imagery, disembodiment, and unity experience.

While it is important to consider that reported SSA/SKAs relate to participants' most powerful experience, results suggest that the SSA/SKA phenomenon is closer in distribution and magnitude to ASCs produced by strong doses of potent psychedelic drugs, particularly those capable of inducing mystical experiences such as psilocybin and DMT, than to any other measured drug and non-drug induced ASC.

### Trait Absorption and TLL as Predictors of SSA/SKA Intensity

Our second hypothesis was tested by performing multiple linear regression between the two independent variables: trait absorption and TLL, as measured with MODTAS and IIPSS, respectively, and each four dependent variables, which present different ways of quantifying the intensity of SSA/SKA: nondual experience, measured with NETI; kundalini awakening, measured with KAS; mystical experience, measured with MEQ30; and altered state of consciousness, measured with the 11D-ASC.

All assumptions of linear regression, including assumptions of normality, were sufficiently met. Since there were four regression models with two predictors each, a Bonferroni multiple comparisons correction was applied to adjust α = 0.05/4 = 0.01.

A statistically significant association between MODTAS and IIPSS, and NETI scores was observed: *F*_(2, 141)_ = 9.67, *p* < 0.001, *R*^*2*^ = 0.12. Both IIPSS (*B* = −0.19, *p* < 0.001) and MODTAS (*B* = 0.29, *p* < 0.001) added significantly to the prediction. The standardised regression coefficients were −0.33 for IIPSS, and 0.39 for MODTAS, suggesting that MODTAS may be a stronger predictor of NETI than IIPSS. However, the two IVs are not sufficient to explain the variance in the dependent variable, as shown by the low value of *R*^*2*^.

The association between MODTAS and IIPSS, and KAS scores was also found to be statistically significant: *F*_(2, 141)_ = 35.74, *p* < 0.001, *R*^*2*^ = 0.34. Both IVs added significantly to the prediction: *B* = 0.23, *p* = 0.006 for IIPSS, and *B* = 0.56, *p* < 0.001 for MODTAS. The standardised regression coefficients were 0.23 and 0.42 for IIPSS and MODTAS, respectively, suggesting that MODTAS may be a stronger predictor of KAS than IIPSS.

Modified Tellegen Absorption Scale and IIPSS significantly predicted MEQ30 scores: [*F*_(2, 143)_ = 23.88, *p* < 0.001, *R*^*2*^ = 0.25]. Modified Tellegen Absorption Scale was a significant predictor (*B* = 0.66, *p* < 0.001), but the IIPSS was not (*B* = −0.07, *p* = 0.40). The standardised regression coefficients were −0.07 and 0.54 for IIPSS and MODTAS, respectively.

Modified Tellegen Absorption Scale and IIPSS significantly predicted 11D-ASC scores: *F*_(2, 143)_ = 34.91, *p* < 0.001, *R*^*2*^ = 0.33. Modified Tellegen Absorption Scale was a significant predictor (*B* = 1.12, *p* < 0.001), but the IIPSS was not (*B* = 0.41, *p* = 0.02). The standardised regression coefficients were 0.21 and 0.43 for IIPSS and MODTAS respectively.

In these models, MODTAS was a better predictor of all outcome variables. Iowa Interview for Partial Seizure-like Symptoms on the other hand, was a significant predictor of all outcome variables with the exception of MEQ30 and 11D-ASC. Furthermore, an increased value of MODTAS predicted an increased score for all DVs, whereas the sign of prediction changed for IIPSS (i.e., negative for NETI and MEQ30). Whilst all four scales used to measure the DVs quantify the intensity of SSA/SKAs, they measure varying aspects of the same experience, which is reflected in the differences between the results of the four regression models.

## Discussion

### Differences Between SKAs and SSAs

The extent to which SKAs differ from SSAs has seldom been explored in psychological literature, and both terms have been used interchangeably to refer to the same experience in past research. While the clear-cut categorisation of such subjective experiences may be problematic, the interchangeability of both terms can be confusing if the experiences they refer to vary, even if only slightly. The hypothesis that SKAs score higher in physical and negative symptoms than SSAs, was therefore introduced to help better identify the subtle differences between both types of spontaneous awakening experiences.

The supposition that SKAs were more likely to produce greater physical and negative effects than SSAs stemmed from the frequent references in transpersonal literature associating subjectively intense, energetic, and often physically challenging awakening experiences to spiritual emergency (Goretzki et al., 2013; St Arnaud and Cormier, 2017; Woollacott et al., 2020). Our results, which indicate that SKAs are significantly more physical than SSAs, are congruent with existing literature on kundalini awakenings, which has frequently alluded to the dominant physical characteristics of this type of awakening experience (Sanches and Daniels, 2008; Goretzki et al., 2013; De Castro, 2015; Lindahl et al., 2017; Lockley, 2019). However, the assumption that SKAs are significantly more negative than SSAs, informed by existing literature alluding to the strong associations between physical and negative symptoms in kundalini awakenings (Lukoff, 1985; Greyson, 1993; Johnson and Friedman, 2008), was not met in this study. It is worth noting, however, that these associations have merely been postulated in existing literature, and that these differences have not, until now, been explicitly laid-out, leaving much room for interpretation. While aiming to address this lack of information, the hypothesis of our exploratory study was therefore also founded on it.

Furthermore, and importantly, a number of items relating to negative experience in the KAS scale also referred to physical symptoms that may not have been considered overwhelmingly negative at the time of the SSA/SKA experience. For instance, item 11: “I've experienced an odd functioning of my reproductive system” (Sanches and Daniels, 2008), may have been a relatively pleasant experience for some. Similarly, item 24: “I've experienced my mind as an uncontrollable incessant flux of ideas or thoughts” (Sanches and Daniels, 2008), may not have been interpreted as particularly negative or challenging to the experiencer at the time of their experience. The inclusion of these and similar items in the KAS negative subscale may therefore be problematic.

It is also worth considering that, whilst a general definition of awakening experiences was provided in the study brief, participants were left to self-determine and label their recalled most powerful awakening experience as either SSA or SKA, and no definition of these individual terms was provided by the authors to facilitate this process. Whilst this was intentional, the subjective interpretation of these terms may have caused slight inconsistencies during the sampling process of both sets of experiences. For instance, participants undergoing spontaneous awakening experiences with strong physical and/or negative symptoms may not necessarily identify with the term SKA if they come from socio-cultural and religious backgrounds that do not identify with Eastern culture and/or philosophy, or if they have not been exposed to the terminology, as may be the case for individuals who have had these experiences outside of a spiritual or religious context. This speculation is partially supported by the reported significant factors that participants felt led them to have the experience, in that yoga practice was more commonly reported in the SKA group compared to the SSA group, and “no discernible trigger” was more commonly reported in the SSA group compared to the SKA group.

Whilst this section aimed as a preliminary exploration of the main differences between SSAs and SKAs, more in depth analysis is warranted, and multi-group confirmatory factor analysis comparing theses experiences with each other and with other altered states experiences will be reported in a future study. Furthermore, future studies should consider using the SES (Goretzki et al., 2014) to better distinguish between individuals experiencing spiritual emergency and those that are not, which could be explored against reported SSA and SKA experiences.

### Effects on Well-Being

The highest scoring items on the NETI, KAS, and MEQ30 scales suggest that the general characteristics of SSA/SKAs are of a predominantly positive nature. Additionally, an overwhelming majority of participants reported that their SSA/SKA had a predominantly positive impact on their well-being in both the short and long-terms, when asked whether their experience was predominantly positive or negative. A higher percentage of participants reporting the positive long-term well-being effects mediated by SSA/SKAs, than those reporting positive short-term well-being effects, suggests that SSA/SKAs may still be perceived as overwhelmingly positive in the long-term, even when the experience was initially challenging. It is also worth noting that over half of our participants reported psychological turmoil or trauma as a significant factor which they believed led them to have the experience, making it the highest reported factor of SSA/SKA in our sample. This is in line with results from existing research (Greyson, 2000; Taylor, 2012, 2015; Woollacott et al., 2020), and further supports the potential for these experiences to yield deep, transformational shifts which can result in long-lasting therapeutic change. Furthermore, the results from our study indicate an increase across all measured spiritual and holistic practices post-SSA/SKA experience, except for the use of psychedelics and entheogens. These activities, including contact with nature, mindfulness, yoga, and meditation, cultivate healthy internal, pro-environmental, and pro-social behaviours such as increased altruism, empathy, trust, confidence, optimism, reduced stress and depression, and better problem solving (Patel et al., 1985; Khalsa et al., 2008; Khanna and Greeson, 2013; Lifshitz et al., 2019). These results, therefore, not only support the supposition that SSA/SKAs mediate overall positive short and long-term effects on well-being, but also that they trigger shifts towards more positive ways of living. These results are consistent with existing studies (Taylor, 2012, 2015; Taylor and Egeto-Szabo, 2017; McGee, 2020).

Mystical experiences (including spiritual awakenings), have been linked to promising improvements in both *subjective* and *objective* states of well-being, as is evidenced by the sustained improvements of treatment-resistant depression (Carhart-Harris et al., 2016), significant reductions in anxiety, hopelessness, and fear of death in patients with life-threatening cancer (Ross et al., 2016), and treatment of treatment-resistant alcohol and tobacco addiction (Green et al., 1998; Garcia-Romeu et al., 2014), mediated by psilocybin-occasioned mystical experiences. Our results support the potential for awakening experiences of a spontaneous nature to occasion deeply therapeutic short and long-term benefits. Our study thus challenges the default pathologisation of spontaneous awakening experiences, addresses the importance for an immediate de-stigmatisation of these experiences within psychiatry, and invites a more holistic, patient-centred approach to researching spiritual and transcendent experiences.

### Relationship to Other ASCs

The observed similarities between the score distributions of SSA/SKA ASCs and all measured non-drug ASCs suggest phenomenological similarities between the groups, supporting the postulation, originally put forward by psychiatrist Ludwig (1966), that all ASCs are phenomenologically similar, whether induced or spontaneous. However, the considerably higher mean scores observed across all subscales in the SSA/SKA sample relative to all measured non-drug ASCs suggests that SSA/SKAs are generally reported as subjectively more intense.

Whilst inferential comparisons were not carried-out between the score distributions of SSA/SKA ASCs and drug-induced ASCs due to a lack of access to raw data from published studies, observed comparisons suggest strong phenomenological similarities between both sets of ASCs. The score magnitude observed across most subscales in the SSA/SKA sample relative to those of all measured drug ASCs, also suggests that SSA/SKAs are more powerful ASCs than all measured drug ASCs, including powerful psychedelic drugs. Particularly striking were the observed similarities, both in magnitude and distribution, between SSA/SKAs and drug-induced ASCs capable of triggering mystical experiences: specifically psychedelic drugs psilocybin and DMT. These results are interesting, not least because the profiles of drugs such as psilocybin and DMT are similar to those of SSA/SKAs in their spiritual outcomes and proposed therapeutic effects (Griffiths et al., 2006, 2008; Carhart-Harris et al., 2016; Ross et al., 2016). It is important to consider, however, that most powerful SSA/SKA experiences were compared to one-off drug and non-drug induced experiences, and a fairer analysis may have been to compare most powerful SSA/SKAs with recalled most powerful drug or non-drug induced ASCs. However, these findings deserve further exploration. The observed phenomenological similarities between SSA/SKA ASCs and psychedelic ASCs, could indicate the potential for research on psychedelic-occasioned mystical experiences to shed light on some of the neurobiological underpinnings and therapeutic potentials of SSA/SKAs, and SSA/SKA research may similarly help inform the study of psychedelic-occasioned mystical experiences.

The observed comparisons indicate a consistent distributional similarity in phenomenology between SSA/SKAs and all measured drug and non-drug ASCs. These overall results propose, therefore, another dimension through which the SSA/SKA phenomenon may be observed. A future study will be undertaken by the authors to statistically compare SSA/SKA ASCs with a range of drug and non-drug ASCs.

### Predictors of SSA/SKAs

Temporal lobe lability and trait absorption are considered two effective predictors for measuring the proclivity for ASC experiences. These have been used together to measure intensity of ASC experiences produced by drugs such as cannabis, and in non-drug induced contexts such as the anechoic dark room chamber, floatation tank, and during Holotropic Breathwork (Luke et al., 2018, 2019). According to our results, TLL and trait absorption positively correlate with the SSA/SKA experience, implying that individuals scoring higher in TLL and absorption are more likely to experience higher intensity SSA/SKAs. However, trait absorption, which has been used exclusively to predict ASC predisposition in psychedelics such as psilocybin (Studerus et al., 2012; Studerus, 2013), LSD (Carhart-Harris et al., 2015; Terhune et al., 2016), MDMA (Hastings, 2006), ayahuasca (Bresnick and Levin, 2006), and DMT (Timmermann et al., 2018), was found to be a better predictor of all SSA/SKA outcomes than TLL.

Indeed, as noted in the Introduction, this is perhaps not wholly surprising, as trait absorption has been found to predict mystical and quasi-mystical experiences produced endogenously in certain sensory-depriving, homeostasis-unbalancing, and trance-inducing contexts (Rock, 2009; Luhrmann et al., 2010; Bronkhorst, 2016; Luke et al., 2018; Glicksohn and Ben-Soussan, 2020), as well as some of the common characteristics of spiritual states associated with SSA/SKAs, such as stronger empathy (Wickramasekera and Szlyk, 2003; Wickramasekera, 2007), stronger flow states (Marty-Dugas and Smilek, 2019), more pronounced creativity (Wild et al., 1995; Manmiller et al., 2005), a stronger attachment to nature and other forms of life (Kaplan, 1995; Brown and Katcher, 1997), feelings of self-transcendence (Cardeña and Terhune, 2014), more pronounced experiences of synaesthesia (Rader and Tellegen, 1987; Glicksohn et al., 1999; Chun and Hupé, 2016), alterations in time-space perception and meaning (Pekala et al., 1985; Kumar and Pekala, 1988), and paranormal beliefs or experiences (Glicksohn, 1990, 2004; Spanos et al., 1993; Glicksohn and Barrett, 2003; Granqvist et al., 2005; French et al., 2008; Parra, 2008; Zingrone et al., 2009; Luhrmann et al., 2010, 2021; Gray and Gallo, 2016; Parra and Gimenez Amarilla, 2016), in drug and non-drug contexts relative to the general population. The differences in measured predictions of SSA/SKA intensity between TLL and absorption are likely due to the fact that each dependent variable used in our study measured slightly different aspects of overall SSA/SKA experiences (i.e., kundalini awakening, nondual experience, mystical experience, ASC).

Whilst these results further our understanding on the relationship between trait absorption, TLL, and ASCs such as SSA/SKAs, it is important to consider that both MODTAS and IIPSS scales are typically used prospectively to measure proclivity for ASC outcomes, and due to the spontaneous nature of SSA/SKAs, this study was not able to follow the typical protocol. The direction of the correlation between TLL and absorption levels, and SSA/SKA experiences, therefore, remains unclear. The spontaneous nature of SSA/SKAs makes the chances for a future prospective study highly unlikely.

Interestingly, when comparing the TLL score distributions from the SSA/SKA sample with those from the published “normal” population sample, SSA/SKA scores were considerably higher, with the largest observed differences present in the high score range. The greater TLL traits in the SSA/SKA sample compared to the published “normal” population sample may suggest a higher tendency for partial seizure-like symptoms typical of TLE among SSA/SKA experiencers. Similarly, these results might suggest a higher likelihood of experiencing SSA/SKA if an individual is predisposed to experiencing partial seizure-like symptoms, typical of TLE.

The observed mean scores across the MODTAS subscales were found to only be slightly higher in the SSA/SKA sample compared to the published “normal” population sample, with the exception of the ASC subscale, where the mean score was more than double that of the “normal” population. This result is not totally unexpected, considering the very nature of the experiences that the ASC subscale measures—as is further evinced by the two highest scoring items (8 and 9): “I think I really know what some people mean when they talk about mystical experiences” and “I can step outside my usual self and experience an entirely different state of being”—which directly refer to mystical experiences. This would suggest that, whilst absorption predicts the intensity of SSA/SKAs, SSA/SKAs are not limited to individuals with high absorption levels.

Inferential comparisons between the SSA/SKA group and the MODTAS and IIPSS published “normal” population groups were not possible due to a lack of access to raw published data, however, these are proposed for a future study.

## Limitations

In addition to the limitations discussed under each section in the above discussion, this study suffers from the following general limitations. First, participants were asked to retrospectively recall their most powerful SSA/SKA, a method which may be vulnerable to error. However, Griffiths et al. (2006), who conducted a study on the reliability of the recall of mystical experiences, suggested that retrospective recall in this context is not as prone to error as in other contexts, which may be explained by the subjective significance of these experiences. Another limitation is that little research had been conducted prior to this study on the experience of spiritual awakenings, and less so on spiritual awakening experiences of a spontaneous nature. Many of our initial assumptions were therefore based on anecdotal considerations. Finally, it is important to consider that Cronbach's alpha scores for 3 of the 11D-ASC subscales were somewhat lower than previously published figures, and internal consistency was therefore not as robust as the authors would have expected. Findings should therefore be interpreted with some caution.

Future studies should be conducted to qualitatively address the phenomenological variances of overall SSA/SKA experiences, their well-being effects and how they compare with psychopathologies capable of producing mystical experiences. In depth comparative tests should also be conducted to understand how SSAs and SKAs correlate with well-being outcomes in both short and long terms. Another important avenue for future research would be to study whether cultural destigmatisation and access to peer support (e.g., spiritual crisis networks) predict better integration of initially challenging SSA/SKA experiences and increase the likelihood of positive outcomes. Finally, a more statistically rigorous comparison between SSA/SKAs and drug-induced ASCs may help us better understand these phenomena, their neurobiological underpinnings, and their potential therapeutic impacts.

## Conclusion

In conclusion, the phenomenological differences between SSAs and SKAs were significant in physical but not in negative experiences, and both personality trait absorption and TLL were positively associated with the intensity of SSA/SKAs, indicating them as good predictors of the overall experience. Furthermore, SSA/SKAs were found to be phenomenologically similar in distribution, but considerably greater in magnitude, than all other measured ASCs. While inferential statistics were not possible between the SSA/SKA ASC and the ASCs produced by psilocybin and DMT, visual comparisons revealed striking similarities between SSA/SKA and psilocybin, and SSA/SKA and DMT, which is in line with recent studies that have pointed to the positive association between the mystical experiences produced by these drugs, and their therapeutic effects. Given the overwhelmingly positive reported effects of SSA/SKAs on well-being in our study, and given the existing literature supporting the potential for spiritual experiences to both treat disorders such as addiction, depression and anxiety, and move people towards increased pro-social and pro-environmental behaviours, more attention from the research community is warranted. This study highlights the importance of recognising SSA/SKAs as valuable experiences that, if properly navigated, carry the potential to positively transform peoples' lives.

## Data Availability Statement

The raw data supporting the conclusions of this article will be made available by the authors, without undue reservation.

## Ethics Statement

The studies involving human participants were reviewed and approved by Dr. Yang Ye, Acting Chair of Ethics—University of Greenwich Departmental Research Ethics Committee. The patients/participants provided their written informed consent to participate in this study.

## Author Contributions

JSC designed and conducted the research, recruited the participants, analysed the data, wrote and reviewed the paper. DL designed the research, analysed the data and reviewed the paper. All authors contributed to the article and approved the submitted version.

## Conflict of Interest

The authors declare that the research was conducted in the absence of any commercial or financial relationships that could be construed as a potential conflict of interest.

## Publisher's Note

All claims expressed in this article are solely those of the authors and do not necessarily represent those of their affiliated organizations, or those of the publisher, the editors and the reviewers. Any product that may be evaluated in this article, or claim that may be made by its manufacturer, is not guaranteed or endorsed by the publisher.

## Abbreviations

ASC: altered states of consciousness
SKA: spontaneous kundalini awakening
SSA: spontaneous spiritual awakening
SSA/SKA: spontaneous spiritual awakening and spontaneous kundalini awakening
TLE: temporal lobe epilepsy
TLL: temporal lobe lability.
