# Supplementary material for: Spontaneous Spiritual Awakenings: Phenomenology, Altered States, Individual Differences, and Well-Being
Source: Front Psychol. 2021 Aug 19;12:720579. doi: 10.3389/fpsyg.2021.720579 (PMC8417526; doi:10.3389/fpsyg.2021.720579)
Supplement: Supplementary file 1 [file Table_1.pdf]

Supplementary data 1. Mean (M) and standard deviation (SD) scores for each of the 76 items of the Kundalini Awakening Scale (KAS; Sanches and Daniels, 2008).

**Items**	***M***	***SD***
I heard sounds such as tones, music, hissing, thunder, drumming, or the sound of cymbals when no such sound was being produced outside my head.	4.46	2.344
I had telepathic experiences.	5.51	1.841
I saw people or objects that weren't materially present.	4.76	2.246
I experienced doing things that seemed to be controlled by another force or intelligence.	5.07	2.059
I experienced light inside my head.	5.54	2.003
I saw auras around people or objects.	4.27	2.27
I sensed unusual cold inside my body, or on my skin.	3.68	2.274
I experienced a spontaneous, involuntary positioning of my limbs.	3.78	2.354
I experienced having odd breathing patterns at times.	4.53	2.125
I experienced an odd functioning of my excretory system without an apparent physical cause.	3.65	2.231
I experienced an odd functioning of my reproductive system.	3.58	2.127
I experienced an elevation of my consciousness.	6.67	0.968
I heard sounds that didn't seem to be perceived by my ears and this had a positive connotation for me.	4.72	2.27

I experienced my breath temporarily become significantly faster.	4.2	1.985
I experienced a shining halo emanating from my head.	3.76	2.162
I experienced a temporary incapability to read.	3.36	2.167
I experienced feelings of some form of energy stored in the genital region.	4.9	2.048
I felt that my mind started to function differently.	6.61	0.855
I experienced dreams of a prophetic nature.	5.44	1.9
I experienced an expansion of my being.	6.53	1.061
I heard sounds that didn't seem to be produced outside my head, and they made me feel comfortable and at peace.	4.57	2.192
I had experiences of elevation and bliss followed by phases of acute depression.	4.78	2.19
I had an experience of union with some divine force or energy.	6.34	1.405
I experienced my mind as an uncontrollable incessant flux of ideas or thoughts.	5.13	1.991
I feared for my mental sanity.	4.12	2.411
I feel that my mind has developed a more effective mode of functioning since my experience.	6.11	1.132
While meditating I felt carried outside my body.	4.86	2.01
I experienced a light which seemed to be associated with my thoughts.	4.45	2.055

I experienced an odd functioning of my digestive system without any apparent physical cause.	3.83	1.986
I experienced a temporary incapability to control my mind.	4.36	2.041
I had experiences of elevation and bliss.	6.47	1.162
I've felt more connected with people since my experience.	5.86	1.456
I feel that I have developed a new channel of communication within me, since my experience.	6.27	1.255
I experienced a temporary incapability to control my body.	4.3	2.166
I experienced seeing people or objects that weren't materially present and this made me feel scared and frightened.	3.07	2.1
I experienced a halo in my head which increased after a spell of prolonged concentration.	3.45	2.115
My consciousness suddenly became wider than it used to be.	6.09	1.453
My consciousness gradually became wider than it used to be.	5.2	1.875
While meditating I felt immersed in a reality outside space.	5.38	1.737
I had sensations of tickling, itching or crawling that moved around in a patterned manner.	4.14	2.255
Agreeable sounds, like music, have become even more pleasurable to me since my experience.	5.65	1.549
Experiencing light inside my head was a pleasant experience for me.	5.36	1.618
I felt that when I woke up my personality descended from a higher realm to a lower one.	4.22	2.05

Seeing lights that didn't seem to be perceived by my eyes was a pleasant experience for me.	5.18	1.739
I experienced an unusual cold in my body moving from place to place.	3.22	1.97
I perceived an aura around my body that stretched itself when I meditated/contemplated.	4.47	2.119
While meditating I felt immersed in a reality outside time.	5.74	1.621
I felt a spiritual ecstasy that could be compared with the bliss of the sexual union.	5.7	1.726
I've become more sensitive to artistic forms of expression since my experience.	5.95	1.453
I experienced the spontaneous involuntary positioning of my fingers.	3.89	2.141
I've become aware of an increase in the area of my peripheral awareness since my experience.	5.66	1.562
I felt very depressed.	3.54	2.313
My everyday consciousness has become slightly elevated since my experience.	6.1	1.385
I experienced an unusual heat in a specific part of my body.	4.75	2.157
I experienced my dreams as filled with light.	4.11	1.839
I had spells of intense absorption without being able to resist them.	5.06	1.857
I had out of body experiences.	4.99	2.015
I feel that my imagination became highly excitable.	5.61	1.625

I became aware of a blissful sensation coming from the spine where it was concentrated.	4.93	1.989
I saw lights that didn’t seem to be perceived by my eyes.	5.14	2.075
My consciousness has become wider than it was before my experience.	6.47	1.073
I felt a blissful sensation in all my nerves.	5.65	1.775
I’m aware of some deep changes in my personality since my experience.	6.45	1.072
When I’m under some intense spells of absorption I feel that my whole personality is expanded.	5.41	1.715
I felt in touch with something measureless.	6.57	0.896
I saw people or objects that weren’t materially present and that have made me feel comfortable and at peace.	4.68	2.076
I experienced smooth involuntary body movements.	4.36	2.161
I experienced a new form of activity in my sexual organs.	4.35	2.05
I felt in touch with something that was nothing and yet was everything.	6.1	1.508
I feel that my imagination has become very vivid since my experience.	5.53	1.5
I've felt humbled by the contact with a transcendent reality.	6.36	1.133
I experienced a feeling of detachment from the world.	5.7	1.768
My sense of touch became more sensitive than it was before my experience.	5.11	1.835

I experienced an unusual cold only in a specific part of my body.	3.14	1.834
I feel that my emotions have magnified since my experience.	5.36	1.697
My experience has made me aware of a more solid reality that makes the rest appear shadowy and unsubstantial.	5.1	1.722
