# Supplementary material for: Spontaneous Spiritual Awakenings: Phenomenology, Altered States, Individual Differences, and Well-Being
Source: Front Psychol. 2021 Aug 19;12:720579. doi: 10.3389/fpsyg.2021.720579 (PMC8417526; doi:10.3389/fpsyg.2021.720579)
Supplement: Supplementary file 2 [file Table_2.pdf]

Supplementary data 2. Mean (M) and standard deviation (SD) scores for each of the 30 items of the Mystical Experience Questionnaire (MEQ30; Maclean et al., 2012).

**Items**	***M***	***SD***
Loss of your usual sense of time.	3.44	1.459
Experience of amazement.	4.37	0.866
Sense that the experience cannot be described adequately in words.	4.34	1.017
Gain of insightful knowledge experienced at an intuitive level.	4.48	0.789
Feeling that you experienced eternity or infinity.	3.92	1.407
Experience of oneness or unity with objects and/or persons perceived in your surroundings.	3.86	1.467
Loss of your usual sense of space.	3.34	1.565
Feelings of tenderness and gentleness.	3.97	1.165
Certainty of encounter with ultimate reality (in the sense of being able to "know" and "see" what is really real at some point during your experience).	4.18	1.122
Feeling that you could not do justice to your experience by describing it in words.	4.29	1.172
Loss of usual awareness of where you were.	2.76	1.721
Feelings of peace and tranquility.	4.07	1.16
Sense of being "outside of" time, beyond past and future.	3.76	1.594
Freedom from the limitations of your personal self and feeling a unity or bond with what was felt to be greater than your personal self.	4.04	1.184
Sense of being at a spiritual height.	4.03	1.337

Experience of pure being and pure awareness (beyond the world of sense impressions).	4.14	1.142
Experience of ecstasy.	4.01	1.284
Experience of the insight that “all is One”.	4.13	1.288
Being in a realm with no space boundaries.	3.57	1.65
Experience of oneness in relation to an “inner world” within.	3.85	1.394
Sense of reverence.	4.03	1.297
Experience of timelessness.	3.8	1.349
You are convinced now, as you look back on your experience, that in it you encountered ultimate reality (i.e., that you “knew” and “saw” what was really real).	4.07	1.149
Feeling that you experienced something profoundly sacred and holy.	4.38	1.109
Awareness of the life or living presence in all things.	4.33	1.132
Experience of the fusion of your personal self into a larger whole.	4.01	1.374
Sense of awe or awesomeness.	4.47	1.023
Experience of unity with ultimate reality.	4.08	1.23
Feeling that it would be difficult to communicate your own experience to others who have not had similar experiences.	4.24	1.063
Feelings of joy.	4.14	1.213
